# Partial Discharge Characteristics and Aging Identification Model of Polymer Insulation Materials in Environmentally Friendly Insulating Liquids Under Electro-Thermal Aging Conditions

**DOI:** 10.3390/polym18070829

**Published:** 2026-03-28

**Authors:** Wenyu Ye, Yixin He, Xianglin Kong, Tianxiang Ding, Xinhan Qiao, Xize Dai, Jiaming Yan

**Affiliations:** 1 School of Electrical Engineering, China University of Mining and Technology, Xuzhou 221116, China; ts24230090p31@cumt.edu.cn (Y.H.); ts25230016a31@cumt.edu.cn (X.K.); ts23230087p31@cumt.edu.cn (T.D.); qiaoxinhan@cumt.edu.cn (X.Q.); yanjiaming888@163.com (J.Y.); 2 School of Electrical Engineering and Computer Science, The University of Queensland, Brisbane, QLD 4072, Australia; xize.dai@uq.edu.au; 3 State Key Laboratory of Power Transmission Equipment Technology, School of Electrical Engineering, Chongqing University, Chongqing 400044, China

**Keywords:** cellulose insulation paper, degree of polymerization, oil–paper insulation, electro–thermal aging, partial discharge, degradation mechanism

## Abstract

Cellulose paper, a natural polymeric dielectric, determines the lifetime of oil–paper insulation systems in transformers, yet its molecular degradation behavior in ester-based insulating media remains insufficiently clarified. This study investigates the electro–thermal aging of cellulose polymer immersed in soybean-based natural ester (SBNE) and palm fatty acid ester (PFAE), with emphasis on depolymerization and its relationship with partial discharge (PD) activity. Accelerated aging experiments were conducted under combined electrical and thermal stress, and the evolution of the degree of polymerization (DP) was measured to quantify polymer chain scission. Phase-resolved PD (PRPD) patterns were recorded during aging, and multi-dimensional statistical features were extracted and reduced using principal component analysis to characterize degradation-sensitive electrical responses. The results show a progressive decrease in DP with aging time in both ester media, accompanied by distinct PD evolution characteristics, indicating different influences of the two esters on cellulose polymer stability. An ensemble learning model integrating multiple classifiers was further employed to identify aging stages based on PD features, achieving reliable discrimination performance. These findings establish a correlation between cellulose depolymerization and dielectric discharge behavior, providing a polymer-centered interpretation of aging mechanisms in ester-based oil–paper insulation systems.

## 1. Introduction

In the context of the green and low-carbon transition of power systems, ester-based insulating oils are progressively replacing conventional mineral oils in power transformers because of their environmental compatibility, high fire safety, and biodegradability [1]. In transformer insulation systems, cellulose paper serves as the primary solid dielectric, and its polymeric integrity fundamentally governs mechanical strength and dielectric reliability. The oil–paper composite insulation system, composed of ester-based oil and cellulose paper, therefore determines the long-term operational stability of transformers [2]. During service, the polymer chains of cellulose insulation paper undergo irreversible scission under combined electrical and thermal stresses, resulting in a continuous reduction in the degree of polymerization (DP), which is widely recognized as the most representative indicator of cellulose aging state [3].

Partial discharge (PD) is a typical precursor phenomenon of insulation degradation and sensitively reflects local defects and electric field distortions within oil–paper systems [4]. Previous studies have systematically investigated PD statistical characteristics and their correlation with aging levels in mineral oil–paper insulation [5,6,7,8]. However, these works mainly focused on mineral oil systems and often under single-stress or natural aging conditions [9]. In contrast, ester-based insulating oils—including natural and synthetic esters—exhibit distinct molecular polarity, dielectric response, and moisture affinity, which significantly influence cellulose degradation pathways and DP evolution [10,11]. Although preliminary studies have reported PD behavior in ester oil–paper systems [12,13], systematic investigations linking electro-thermal aging, DP degradation of cellulose paper, and PD feature evolution across different aging stages remain limited. In this study, we address this gap by directly correlating cellulose polymer degradation (degree of polymerization, DP) with statistical features extracted from phase-resolved PD patterns, providing a polymer science–based interpretation of insulation aging and a framework for evaluating cellulose structural integrity under electro–thermal stress.

From a diagnostic perspective, PD analysis has evolved from conventional φ–q–n statistical diagrams to data-driven pattern recognition approaches incorporating machine learning algorithms [4,14]. While such models achieve reliable performance in mineral oil systems, their direct application to ester-based insulation often leads to reduced accuracy due to differences in PD amplitude distribution, phase characteristics, and statistical separability [15]. Therefore, establishing PD feature–aging stage relationships specifically associated with cellulose DP degradation in ester oil environments remains a critical challenge.

To address these issues, this study conducts synchronized accelerated electro-thermal aging and PD monitoring experiments, with particular emphasis on the DP evolution of cellulose insulation paper in two ester-based insulating oils. Large-scale PD datasets are acquired throughout the aging lifecycle, and multidimensional statistical features are extracted and reduced using principal component analysis to construct a DP-correlated PD feature fingerprint library. Four classical algorithms—SVM, RF, ANN, and KNN—are further integrated through a voting-based fusion strategy to enhance robustness in aging stage recognition. The results demonstrate that the proposed feature system effectively captures DP-dependent aging characteristics of cellulose insulation, providing material-oriented insight and technical support for the condition assessment of ester-based transformer insulation systems.

## 2. Experimental Procedure and Results Analysis

### 2.1. Experimental Procedure

#### 2.1.1. Sample Preparation and Pre-Treatment

Electro–thermal aging tests were carried out as the initial stage of this study. Two ester-based insulating oils were selected, including a soybean-based natural ester (SBNE) insulating oil supplied by Henan Jiuyu Sipai Electric Power Technology Co., Ltd., Zhengzhou, China, and a palm fatty acid ester (PFAE) insulating oil provided by Shanghai Sakura Chemical Research & Technology Co., Ltd, Shanghai, China. SBNE and PFAE oils were selected for this study due to their widespread use in transformer applications. As natural ester oils, both are recognized for their excellent environmental performance, including biodegradability and low toxicity, making them highly suitable for environmentally friendly transformer systems. SBNE oil, derived from soybean, and PFAE oil, derived from palm fatty acids, are both commonly used in industrial-scale transformers, with extensive research into their dielectric properties, aging behaviors, and performance in real-world applications. Their key physicochemical parameters are listed in Table 1. Before aging, both insulating oils were treated by vacuum filtration at 60 °C to eliminate residual moisture and suspended contaminants. Kraft insulation paper, a cellulose-based polymeric material composed of long-chain macromolecules, was employed as the solid dielectric material, with a nominal thickness of 0.3 mm and a density of 1.2 g/cm^3^. The paper was machined into circular samples with a diameter of 80 mm for subsequent experiments.

Before the experiments, the insulating oils and cellulose-based insulation papers were preconditioned according to the procedures reported in the literature [16,17]. The insulating oils were dehydrated and degassed at 90 °C under a vacuum of 50 Pa for 48 h, while the insulation paper specimens were dried at the same temperature for 48 h to reduce the moisture content to below 1%. Subsequently, the dried paper samples were immersed in the corresponding insulating oils and maintained at 60 °C under a vacuum of 50 Pa for 24 h to ensure complete oil impregnation and removal of entrapped air within the paper structure. Prior to experimental use, the insulating oils were further purified by vacuum filtration at 60 °C to eliminate residual moisture and particulate contaminants.

The moisture content of the insulating oils was monitored throughout the aging process, as it is an intrinsic factor affecting both partial discharge and breakdown behavior. For the PFAE oil, the moisture content at eight aging stages (0 h, 12 h, 24 h, 72 h, 168 h, 312 h, 504 h, and 768 h) was 237.4, 253.5, 403, 451.5, 518, 1065, 1303.5, and 1756.48 ppm, respectively. For the SBNE oil, the corresponding values were 154.6, 334, 453, 439, 880, 318, 465, and 352 ppm. These data indicate that the moisture content in the oils evolved dynamically during the electro–thermal aging process and differed between the two systems. It should be noted that the moisture content in the oils is not a fully controllable experimental variable, but rather reflects the environmental and operational conditions of the insulation system. Therefore, the observed differences in partial discharge and breakdown characteristics between SBNE and PFAE oils should be interpreted as behavior under the current experimental conditions, rather than an intrinsic performance ranking of the two insulating oils.

#### 2.1.2. Electro–Thermal Aging Procedure

**Figure 1:**
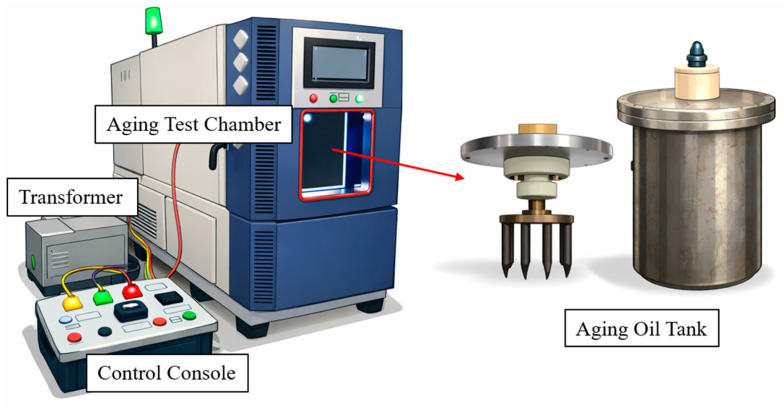
Aging Oil Tank and Experimental Platform.

To reproduce the oil-impregnated insulating paper structure of practical transformers, the preconditioned insulating oils and cellulose insulation papers were combined at a mass ratio of 10:1 and loaded into a sealed electro–thermal aging oil tank, with 7000 g of insulating oil and 700 g of cellulose insulation paper used in each system. The experimental setup is illustrated in Figure 1. A multi-needle-to-plate electrode configuration incorporating ten needle tips was installed inside the tank, and the corona discharges generated by the needle electrodes acted simultaneously on multiple regions of the cellulose insulation paper, thereby ensuring a more uniform electrical stress distribution during aging. In normal operation, the temperature of oil-immersed transformers typically does not exceed 100 °C; therefore, in accordance with IEC 60216-1:2013 [18], the aging temperature was selected to be higher than the maximum allowable operating temperature while avoiding the introduction of non-representative degradation mechanisms, and was determined based on the thermal aging equivalence relationship:

(1) $$t=t_{0}e^{-\alpha (\theta -\theta_{0})},$$

In this expression, *t* denotes the expected service life of the insulation material at the actual operating temperature *θ*, *t*_0_ corresponds to the lifetime at the reference temperature *θ*_0_ = 80 °C, and α is the thermal aging coefficient, which was set to 0.1155. Based on this relationship, thermal aging at 130 °C for 768 h is equivalent to approximately 28 years of operation at 80 °C. Consequently, an accelerated aging temperature of 130 °C was selected for the present study, which is comparable to the service life of practical power transformers. The aging voltage was established by progressively increasing the applied voltage on the oil-impregnated insulating paper tank while continuously monitoring partial discharge activity using a dedicated detection system, and the voltage corresponding to the initial onset of partial discharge was defined as the aging voltage. To ensure stable partial discharges in the oil–paper insulation system, the applied voltage was gradually increased, and a partial discharge detection system was used to determine the corona inception voltage under the multi-needle–plate electrode configuration. While maintaining a sufficient safety margin from the breakdown voltage, an applied voltage of 6 kV, slightly higher than the measured inception voltage, was intentionally selected. This voltage level ensures stable and repeatable discharges during long-term aging experiments while avoiding catastrophic failure of the samples. The oil tank was then transferred to the aging chamber, where electro–thermal aging was carried out at 130 °C under a steady voltage of 6 kV. The electro–thermal aging platform, schematically shown in Figure 1, comprised an aging chamber, a high-voltage transformer, and a control console, allowing accurate regulation of temperature, voltage, and operating conditions. Throughout the aging period, samples were periodically extracted at 0, 12, 24, 72, 168, 312, 504, and 768 h for the measurement of characteristic parameters.

#### 2.1.3. Partial Discharge Measurement Procedure

**Figure 2:**
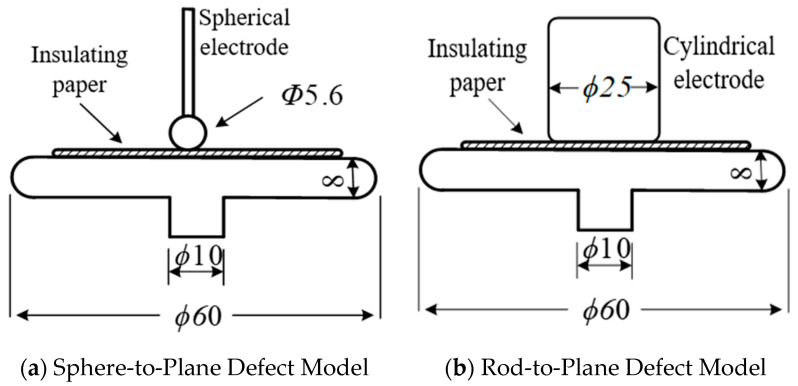
Oil-paper insulation defect model diagram.

In accordance with the experimental design recommendations specified in CIGRE Technical Brochure 662 (2016) [19], following each aging stage, the insulating oils and paper samples were subjected to PD measurements. Two defect models of oil-impregnated insulating paper, namely sphere-to-plane and rod-to-plane, were constructed to simulate the actual air-gap and surface discharges occurring in transformer oil-impregnated insulating paper, as illustrated in Figure 2a and Figure 2b, respectively.

**Figure 3:**
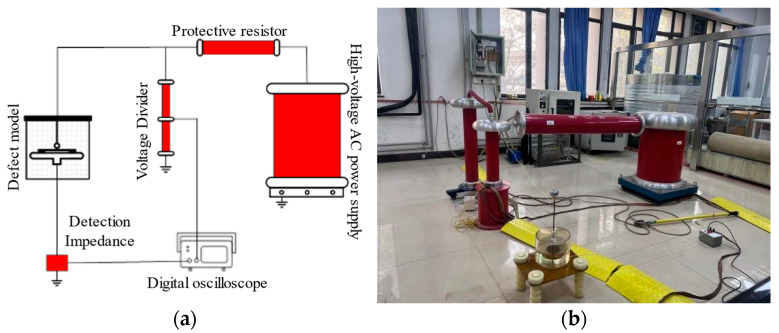
Schematic diagram and photograph of the partial discharge measurement setup for the oil–paper polymeric insulation system. (**a**) Schematic diagram of the wiring con-figuration for partial discharge measurements. (**b**) Photograph of the partial discharge experimental setup.

During the experiments, the sphere-to-plane defect model was used to determine the inception voltage of the insulation paper and to acquire PD signals under a stable applied voltage. To account for the dielectric strength of the cellulose insulation paper and the actual inter-turn insulation conditions of transformers, three layers of circular paper specimens were stacked as the test sample, with each layer having a thickness of 0.3 mm. In contrast, the rod-to-plane defect model was employed for breakdown voltage testing of the oil-impregnated insulating paper. For breakdown tests, a single layer of aged cellulose insulation paper specimen was placed in the electrode gap for each measurement. To systematically investigate the evolution of insulation performance of oil–paper insulation materials at different aging stages, a PD detection system based on the pulsed current method was employed to acquire discharge signals from a series of samples with varying degrees of aging. The schematic diagram and photograph of the experimental setup are shown in Figure 3a and Figure 3b, respectively.

The core components and key parameters of the system are as follows. The high-voltage AC source provided a continuously adjustable voltage from 0 to 50 kV. The test transformer had a rated voltage of 50 kV and a rated capacity of 50 kVA, ensuring stable voltage output with minimal waveform distortion within its rated range. A protective resistor R with a value of 5 kΩ was used, the main function of which was to limit the short-circuit current in the circuit and to prevent excessive surge currents from damaging the high-voltage testing equipment in the event of sample breakdown. The detection unit employed a laboratory-developed RLC-type detection impedance, which was precisely designed and calibrated to enable efficient and accurate extraction of weak discharge pulses. A high-performance digital oscilloscope was utilized for signal recording, with an analog bandwidth of up to 400 MHz and a maximum sampling rate of 20 GS/s, sufficient to capture and digitize the nanosecond-scale discharge pulses without distortion.

Prior to the formal PD measurements, the background noise of the testing environment was first acquired. After all experimental connections were correctly established, fresh ester insulating oil that had not been previously used was poured into a clean test container, ensuring that the oil fully immersed the electrode structure. In this state, no test samples were placed in the container, and the entire high-voltage circuit remained unenergized. The inherent background noise signals were then collected and recorded using the detection impedance and oscilloscope system. The recorded noise data were preliminarily analyzed, and the maximum and average amplitudes were identified to serve as reference values for subsequent data processing. These noise benchmarks provided a basis for effectively separating and identifying real PD pulses from mixed signals.

Following the background noise measurement, the PD characterization of the test samples was conducted. Aged cellulose insulation paper specimens from specific aging periods were carefully placed on the surface of the ground electrode within the test container. Once prepared, a sinusoidal AC voltage was gradually applied to the sample in a smooth and controlled manner. The voltage at which the first discernible PD pulses, exceeding the background noise level, were observed on the oscilloscope was recorded as the inception voltage of the sample. Subsequently, a constant-voltage method was employed to perform long-term PD measurements. Starting from the measured inception voltage, the applied voltage was further and gradually increased until the PD pulses recorded by the oscilloscope stabilized in both amplitude and frequency. Once the stable discharge stage was reached, the voltage was maintained constant, and PD signals were continuously acquired using the detection impedance and oscilloscope system. To ensure statistical validity and repeatability, PD signals were collected for each sample over 500 cycles of the power frequency.

### 2.2. Morphological Evolution of Cellulose Insulation Paper During Aging Experiments

**Figure 4:**
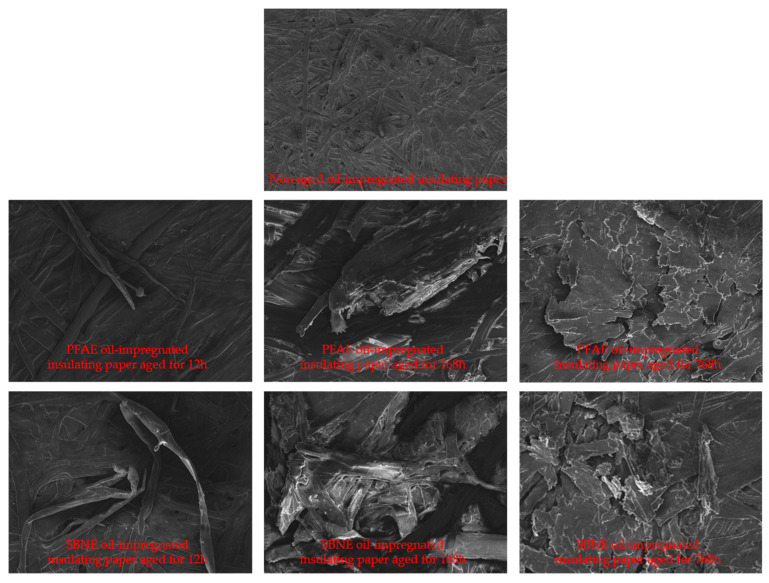
SEM Morphologies of Oil-Impregnated Insulating Paper at Different Aging Stages.

To further elucidate the microstructural degradation behavior of oil-impregnated insulating paper under electro–thermal aging, scanning electron microscopy (SEM) was employed to examine its morphological evolution at different aging stages. Under combined electrical and thermal stresses, cellulose polymer chains gradually undergo aging and chain scission, leading to progressive deterioration of the fiber network structure and consequently affecting the macroscopic insulation performance. The SEM morphologies of oil-impregnated insulating paper aged in two ester oils at different aging stages are shown in Figure 4. For the unaged sample, the fiber structure appears uniform and well organized, with clearly defined contours and relatively consistent fiber diameters. No obvious damage or degradation features are observed, indicating that the cellulose network remains intact. After 12 h of electro–thermal aging, slight fiber separation and localized fracture features begin to appear. At this stage, the surface structure becomes relatively more compact. This phenomenon may be attributed to the formation of shorter cellulose chains during early degradation, which may migrate and fill micro-voids between adjacent fibers. Additionally, structural rearrangement or partial recrystallization in amorphous cellulose regions may temporarily densify the fiber network. After 168 h of aging, the degradation becomes more evident, with large areas of structural damage accompanied by fiber distortion and fracture. With further aging up to 768 h, the morphology becomes highly deteriorated, exhibiting flocculent and fragmented structures. At this stage, the fiber network is severely disrupted, indicating extensive cellulose chain scission and a significant loss of structural integrity.

A comparison of the two oil-impregnated systems shows that, at the same aging duration, samples aged in SBNE insulating oil exhibit more severe morphological damage than those aged in PFAE insulating oil, suggesting a faster degradation rate of cellulose in the SBNE environment.

### 2.3. Analysis of Degree of Polymerization of Cellulose Insulation Paper During Aging Experiments

**Figure 5:**
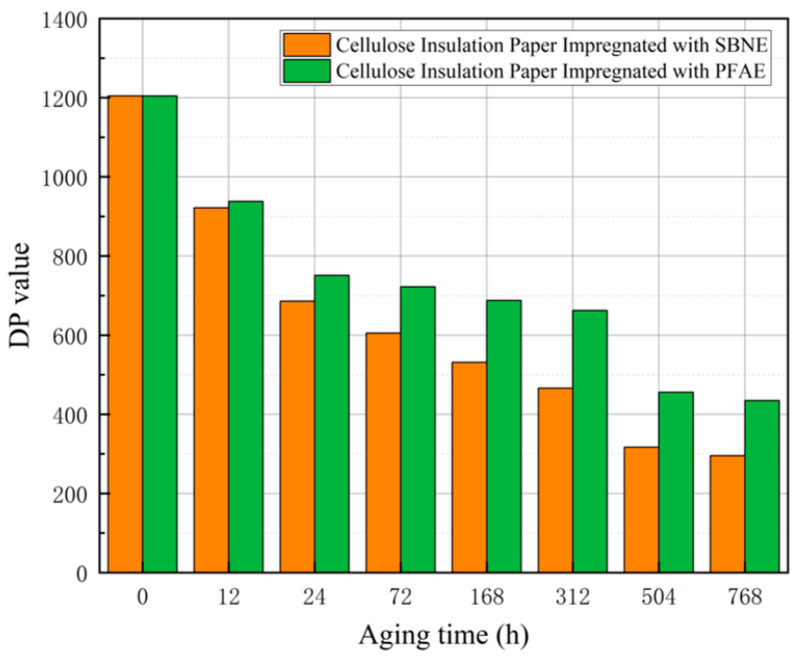
DP value during the aging process.

The SEM observations described above reveal the progressive deterioration of the fiber network structure of cellulose insulation paper during electro–thermal aging. To further quantify the molecular degradation underlying these morphological changes, the degree of polymerization (DP) of cellulose was measured as a key indicator of the aging state of the insulating paper. DP directly reflects the average length of cellulose molecular chains and is closely associated with the mechanical strength and dielectric performance of the material. After completion of the aging experiments, the DP of insulation paper immersed in the two insulating oils was measured immediately, and its evolution during the thermal aging process is illustrated in Figure 5. To ensure the repeatability and reliability of the measurements, each DP value was measured three times and the average value was used in the analysis. The corresponding standard deviations were also calculated, and all standard deviation values were within 5, indicating good measurement consistency. According to the evolution of the degree of polymerization (DP), the electro–thermal aging process of cellulose insulation paper can be distinctly divided into three characteristic stages, reflecting different degradation kinetics of the polymer chains. In the initial stage (0–24 h), a pronounced and rapid decline in DP is observed, with reductions of 43% and 37.6% for the paper aged in SBNE and PFAE, respectively. This sharp decrease indicates intensive chain scission of cellulose macromolecules, primarily attributed to the preferential degradation of the less ordered amorphous regions, where glycosidic bonds are more susceptible to hydrolytic and thermo-oxidative cleavage. As aging progresses into the intermediate stage (24–504 h), the DP continues to decrease but at a significantly reduced rate. This transition suggests that, after the rapid consumption of vulnerable amorphous segments, the degradation process becomes increasingly governed by the gradual breakdown of more stable crystalline domains, leading to slower depolymerization kinetics. By the final stage (504–768 h), the DP approaches a quasi-equilibrium state, stabilizing at 283.9 and 296.1, respectively. The plateau behavior implies that the residual cellulose network consists predominantly of shorter yet relatively stable molecular chains, and further chain scission occurs at a limited rate.

Throughout the entire aging process, the consistently higher DP values observed in PFAE indicate a comparatively stronger suppression of cellulose depolymerization, demonstrating its enhanced capability to retard the molecular degradation of cellulose insulation paper under electro–thermal stress. Such stage-dependent depolymerization behavior directly influences the dielectric integrity of the oil–paper system and provides a molecular basis for interpreting the evolution of partial discharge characteristics during aging.

Kinetic equations are commonly employed to describe and analyze the evolution of the degree of polymerization and the aging rate of insulation paper. In this study, a second-order kinetic model [20] was adopted, as expressed in Equation (2), where ${DP}_{0}$ and ${DP}_{t}$ denote the degree of polymerization of unaged insulation paper and that after a thermal aging time *t*, respectively. The parameter $k_{1}$ represents the initial aging rate, while $k_{2}$ characterizes the attenuation rate of $k_{1}$ with aging progression. By fitting the experimental DP data using the proposed kinetic model, the initial aging rate $k_{1}$, and the aging rate decay coefficient $k_{2}$ were determined for insulation paper immersed in the two ester-based insulating oils.

(2) $$\frac{1}{DP_{t}}-\frac{1}{DP_{0}}=\frac{k_{1}}{k_{2}}(1-e^{-k_{2}t}),$$

Based on the fitted second-order kinetic parameters, the initial aging rate of insulation paper immersed in SBNE oil ($k_{1}$ = 3.11 × 10^−3^) is higher than that in PFAE insulating oil ($k_{1}$ = 2.81 × 10^−3^), as summarized in Table 2, indicating more pronounced cellulose chain scission and faster degradation during the early aging stage in the soybean ester system. In contrast, the aging rate decay coefficient $k_{2}$ of cellulose insulation paper impregnated with PFAE oil (8.0647 × 10^−6^) exceeds that of the cellulose paper impregnated with soybean-based ester oil (4.9138 × 10^−6^), suggesting that the aging rate in the PFAE system decreases more rapidly with time and transitions earlier into a relatively slow degradation regime.

To evaluate the reliability of the kinetic fitting, statistical goodness-of-fit metrics were calculated. For the SBNE oil-impregnated paper, the second-order kinetic model yields an R^2^ of 0.9085 and an RMSE of 0.00024042, while for the PFAE oil-impregnated paper, the corresponding R^2^ and RMSE are 0.7777 and 0.00020715, respectively. These values indicate that the model captures the overall trends of polymerization degree decay well, particularly for the SBNE system, and provides a reasonable quantitative description of cellulose degradation kinetics for both oils.

Overall, the aging rate of insulation paper reaches its maximum at the initial aging stage and gradually declines thereafter, which is consistent with the observed evolution of the degree of polymerization. Throughout the entire aging process, the aging rate of insulation paper in SBNE oil remains higher than that in PFAE oil, resulting in a lower polymerization degree. These results demonstrate that PFAE insulating oil exhibits superior capability in suppressing early-stage rapid aging and mitigating long-term degradation of cellulose insulation.

### 2.4. Analysis of Cellulose Insulation Paper Characteristics During Partial Discharge Experiments

#### 2.4.1. Analysis of Discharge Voltage

- Inception Voltage

**Figure 6:**
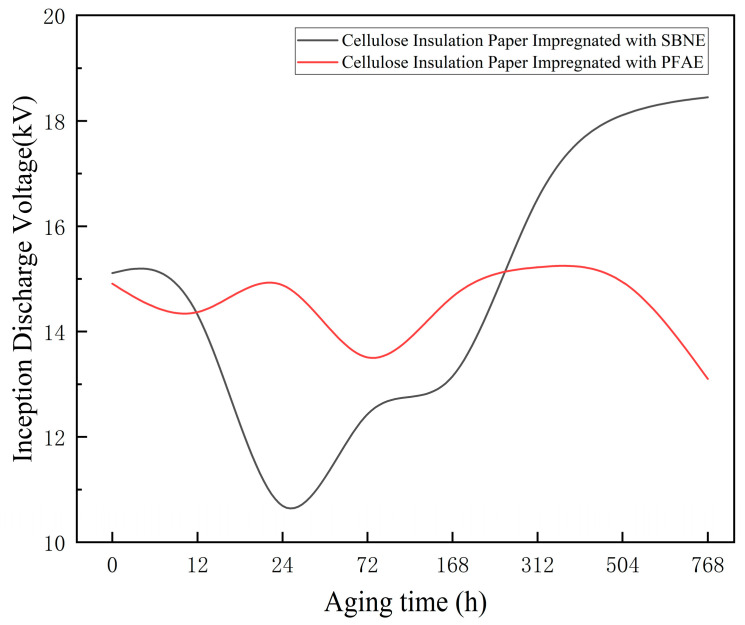
Trend of Inception Voltage of Oil-Impregnated Insulating Paper.

During the PD measurements, the inception voltages of the insulation paper samples immersed in the two types of ester oils at different aging stages were measured and recorded. To ensure the reliability and repeatability of the measurements, the inception voltage at each aging stage was measured ten times, and the average value and the corresponding standard deviation were calculated. The average inception voltages and standard deviations at each aging stage are summarized in Table 3. Figure 6 illustrates the variation in the inception voltage with increasing aging severity for the two types of ester oil–paper samples, where the variation trend is analyzed based on the average values.

As shown in Table 3 and Figure 6, the inception voltage of the cellulose insulation paper in SBNE oil exhibited a non-monotonic trend with aging time, initially decreasing and then increasing, and demonstrated improved discharge suppression capability after long-term aging compared with the initial state. In contrast, the inception voltage of the cellulose insulation paper in PFAE oil remained relatively stable throughout the entire aging period, with a slight decrease observed after prolonged aging.

The experimental results indicate that during the early aging stage, the variation in inception voltage for cellulose insulation paper impregnated with PFAE oil was significantly smaller than that for cellulose insulation paper impregnated with SBNE oil, reflecting the superior stability of the synthetic ester under initial aging conditions. Under long-term aging, however, the inception voltage of the cellulose insulation paper in SBNE oil was notably higher than that in PFAE oil, suggesting that aging products in the natural ester oil play a positive role in inhibiting the onset of partial discharge. This behavior is consistent with the observed discharge suppression advantage of natural ester oil in the later stages of aging.

- Breakdown Voltage

**Figure 7:**
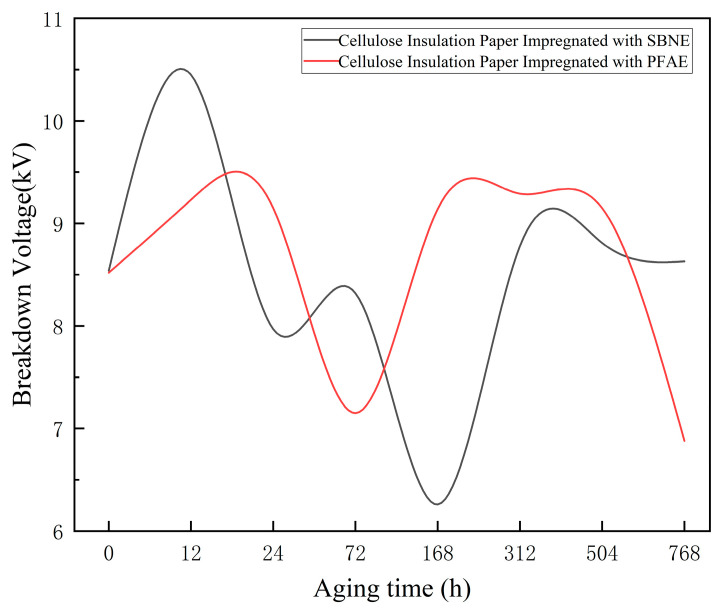
Trend of Breakdown Voltage of Oil-Impregnated Insulating Paper.

During the PD measurements, the breakdown voltages of the insulation paper samples immersed in the two types of ester oils at different aging stages were simultaneously measured and recorded, and the average values were subsequently calculated. To ensure the reliability and repeatability of the measurements, the breakdown voltage at each aging stage was measured ten times, and both the average value and the corresponding standard deviation were calculated. The average breakdown voltages and standard deviations of each oil–paper sample at different aging stages are summarized in Table 4. Figure 7 illustrates the variation in breakdown voltage with increasing aging severity for the two types of ester oil–paper samples, where the trends are analyzed based on the average values.

As shown in Table 4 and Figure 7, the breakdown performance of cellulose insulation paper in SBNE oil was more sensitive to aging, with the intermediate aging stage representing a critical window during which insulation deterioration was most pronounced. In contrast, the breakdown voltage of the cellulose insulation paper in PFAE oil remained relatively stable during the intermediate stage, indicating that the synthetic ester exhibits stronger inhibition of aging-induced breakdown channels at this stage. However, under long-term aging conditions, both oils experienced varying degrees of insulation performance degradation.

The experimental results indicate that cellulose insulation paper undergoes differentiated degradation behaviors when aged in different ester media. In the SBNE system, the higher moisture content and the accumulation of polar aging by-products promote hydrolytic cleavage of cellulose molecular chains, facilitating the formation of defect sites that can evolve into electrical breakdown channels. In contrast, the relatively stable molecular environment provided by PFAE oil mitigates the depolymerization rate of cellulose during the early and intermediate aging stages, resulting in comparatively higher dielectric strength of the oil–paper system. However, as aging progresses and decomposition products accumulate, the protective effect gradually diminishes, leading to an accelerated decline in breakdown voltage. Overall, the stage-dependent evolution of electrical properties is closely associated with the depolymerization behavior of cellulose polymer, which is governed by the polarity of the surrounding oil, its moisture solubility characteristics, and the generation of aging by-products.

#### 2.4.2. Analysis of PRPD Pattern Characteristic Parameters

- Construction of PRPD Patterns

In the statistical analysis of PD patterns, phase-resolved partial discharge (PRPD) maps are widely used due to their simplicity of implementation and clear physical significance [21]. Based on PRPD, various characteristic maps, such as histograms, scatter plots, and grayscale maps, can be generated [22]. The PRPD pattern within a single power frequency cycle is composed of the apparent discharge quantity Q, the number of discharges n, and the discharge phase *φ*. Due to the inherently stochastic nature of PD, parameters measured over multiple power frequency cycles are often aggregated into a single cycle to highlight statistical regularities. The constructed PRPD maps can reflect the relationships among discharge quantity, discharge phase, and the number of discharges in oil–impregnated paper samples. Based on the PRPD maps, the partial discharge characteristics at different aging stages of the oil-impregnated insulating paper can be preliminarily assessed [23].

- Extraction of PRPD Characteristic Parameters

By constructing the phase distributions of the maximum discharge magnitude *Q*_max_ − *φ*, the average discharge magnitude *Q_ave_* − *φ*, and the discharge count *n* − *φ*, a total of 34 statistical characteristic operators were extracted to quantitatively describe the PRPD patterns and to distinguish different discharge behaviors. According to the characteristic parameter extraction scheme, the above PRPD patterns were divided into positive and negative half-cycles based on the phase angle, and the characteristic parameters were calculated separately for each half-cycle. The detailed definitions of the statistical characteristic operators are listed in Table 5.

The above-mentioned feature parameters comprehensively characterize the partial discharge information in the patterns from different perspectives. A total of 34 statistical features were extracted, including kurtosis (*Ku*), skewness (*S_k_*), peak number (*Pe*), discharge factor (*Q*), correlation coefficient (*CC*), modified cross-correlation coefficient (*m_cc_*), impulse factor (*I*), margin factor (*L*), and centroid frequency (*FC*).

A total of 34 partial discharge features were extracted from the PRPD patterns. Statistical analysis indicates that, in the maximum discharge magnitude Q–F phase distribution of both ester oil-impregnated insulating paper, the skewness (*S_k_*), modified cross-correlation coefficient (*m_cc_*_1_), margin factor (*L*), centroid frequency (*FC*), and impulse factor (*I*) exhibit a pronounced correlation with the aging stage.

**Figure 8:**
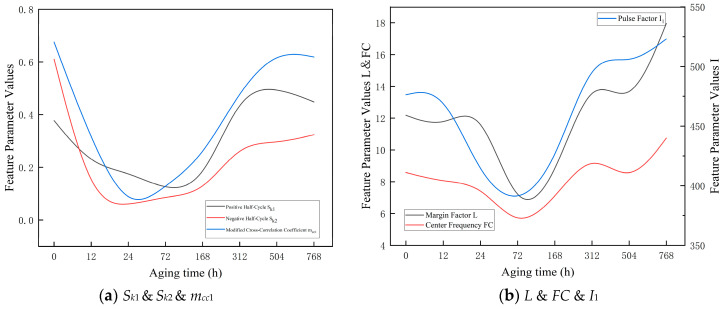
Trend of Characteristic Parameters of Cellulose Insulation paper Impregnated with SBNE.

As shown in Table 6 and Figure 8, both the skewness (*S_k_*) and the modified cross-correlation coefficient (*m_cc_*_1_) of the positive and negative half-cycles in the PRPD patterns of SBNE insulating oil exhibit a pronounced decreasing trend followed by a gradual recovery. Before 168 h of aging, the generation of low-molecular-weight polar by-products and trace moisture in the insulating oil weakens the discharge intensity in the negative half-cycle and reduces the discharge symmetry between the positive and negative half-cycles. The decline in mcc indicates a transition of the discharge pattern from a concentrated mode to a more dispersed one, accompanied by enhanced discharge randomness. After 168 h of aging, as aging by-products accumulate to a critical concentration, the increased amount of suspended particulate matter in the oil induces electric field distortion, which may be associated with intensified oil-gap discharges, local electric-field distortion, and possible stabilization of preferential discharge paths. Consequently, the discharge activity remains at a relatively high and stable level. The recovery and sustained high values of mcc indicate that the phase distribution of partial discharges becomes more concentrated, corresponding to the formation of stable microscopic discharge paths within the oil-impregnated insulating paper, along with carbonization traces on the surface of the insulation paper.

Meanwhile, the margin factor (*L*) shows an initial increasing trend followed by stabilization. During the early aging stage (0–168 h), the accumulation of polar degradation products and moisture enhances partial discharge activity, resulting in an increase in L. After 168 h, the formation of stable oil-gap discharges and continuous conductive channels causes L to remain at a relatively high level.

The impulse factor (*I*) exhibits a rise–fall trend. During the early and middle aging stages (up to 504 h), the enhancement of discharge pulse amplitudes due to the accumulation of aging by-products leads to an increase in I. In the late aging stage (768 h), as discharge activity approaches saturation and the insulation structure further deteriorates, *I* decreases accordingly.

The centroid frequency (*FC*) shows an overall decreasing trend, indicating a gradual shift in discharge frequency components toward lower frequencies with aging. This behavior reflects the progressive degradation of the dielectric properties of the oil-impregnated insulating paper caused by the accumulation of aging by-products.

**Figure 9:**
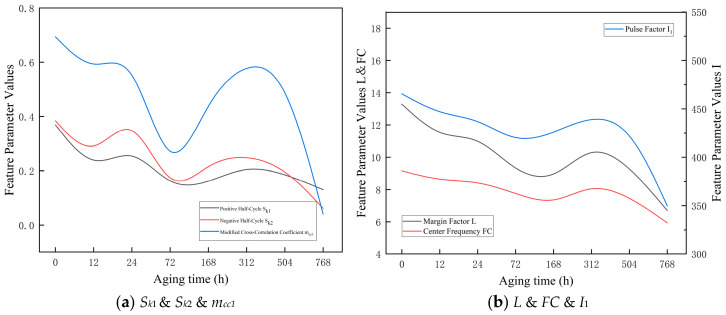
Trend of Characteristic Parameters of Cellulose Insulation Paper Impregnated with PFAE.

As shown in Table 7 and Figure 9, the skewness (*S_k_*) and the modified cross-correlation coefficient (*m_cc_*) of both the positive and negative half-cycles in the PRPD patterns of PFAE insulating oil exhibit a fluctuating trend characterized by an initial decrease, followed by a recovery, and then a subsequent decline.

During the early aging stage (0–72 h), trace impurities and the release and migration of dissolved gases in the PFAE oil induce dynamic adjustments of the local electric field. The rapid decrease in mcc indicates a transition of the discharge pattern from a relatively concentrated state to a highly dispersed mode, suggesting that the insulation system is undergoing an unstable adjustment phase. In the mid-to-late aging stage (168–504 h), the *S_k_* values of both half-cycles tend to stabilize, as the accumulation of oxidation products from ester molecules forms a relatively stable colloidal suspension. This process partially homogenizes the electric field distribution. The recovery of mcc indicates that partial discharge activity gradually concentrates within specific phase intervals. During this stage, the overall discharge intensity remains relatively stable, although the insulation system has already entered a cumulative degradation process. In the late aging stage (768 h), the *S_k_* values of both half-cycles decrease further to lower levels, indicating that the PFAE insulating oil has entered the final stage of aging. Significant oil decomposition occurs, producing a large number of conductive aging products, resulting in more numerous and widely distributed partial discharge points without obvious phase concentration. The sharp decline in mcc demonstrates that multiple randomly distributed weak discharges are present in the oil-impregnated insulating paper, indicating that the insulation is approaching failure.

Meanwhile, the margin factor (*L*) exhibits a fluctuation trend of initial increase, subsequent stabilization, and slight decline. During 0–72 h, the dynamic release of trace impurities and dissolved gases causes local electric field adjustments, leading to an increase in *L*. In the 168–504 h stage, the formation of a relatively stable colloidal suspension homogenizes the electric field, maintaining L at a relatively steady level. After 768 h, significant oil decomposition produces numerous conductive aging products, increasing the number and spatial distribution of partial discharge points, resulting in a slight decrease in *L*.

The impulse factor (*I*) shows a rise–fall trend. During the mid-aging stage, the accumulation of oxidation products enhances discharge intensity, leading to elevated I values. In the late aging stage, as the insulation system reaches the final cumulative degradation phase, discharge becomes more dispersed and weaker, and I correspondingly decreases.

The centroid frequency (*FC*) exhibits an overall decreasing trend, indicating that discharge activity gradually shifts toward lower frequencies with aging. This behavior reflects the progressive breakdown of the molecular structure of PFAE oil and the increase in aging by-products, resulting in an overall slowdown of discharge activity.

## 3. Pattern Recognition of Aging Stages for Cellulose Insulation Paper

### 3.1. Dimensionality Reduction in Feature Parameters

Redundant information may exist among the feature operators, which can lead to excessive information redundancy. Directly inputting these features into a classifier not only consumes a large amount of memory but also adversely affects recognition accuracy and reduces the processing speed of the classifier. Therefore, dimensionality reduction is required to extract feature parameters with strong discriminative capability and minimal redundancy.

Principal Component Analysis (PCA) is a classical unsupervised linear dimensionality reduction method [24]. Its core idea is to perform an orthogonal linear transformation on the original correlated variables, converting them into linearly independent principal components that are sorted according to the explained variance, thereby retaining the maximal amount of original feature information. The objective of PCA is to reduce the data dimensionality while preserving the inherent structure of the data as much as possible, facilitating subsequent visualization, compression, and analysis [25].

This method is based on the statistical principle that variance measures information content. By minimizing the mean squared error, PCA constructs an optimal orthogonal transformation to achieve effective dimensionality reduction. Geometrically, PCA enables a clearer representation of the data in a lower-dimensional space; algebraically, it establishes the covariance matrix, solves for eigenvalues and eigenvectors, determines the number of principal components based on the cumulative contribution rate, and projects the original features onto a new coordinate system to form new feature parameters.

The derivation procedure of PCA can be briefly described as follows:

1. Data Standardization: Since the magnitudes of different features may vary, the data are first standardized to have a mean of 0 and a variance of 1.

2. Covariance Matrix Calculation: The covariance matrix describes the linear relationships among the features. For a dataset X consisting of n samples and p features, the covariance matrix *C* can be expressed as:


(3) $$C=Var(X)=\Lambda =\left[\begin{array}{cccc} \lambda_{1} & & & \\ & \lambda_{2} & & \\ & & \ldots & \\ & & & \lambda_{n} \end{array}\right],$$

3. Eigenvalue Decomposition: The covariance matrix *C* is subjected to eigenvalue decomposition to obtain the eigenvalues and their corresponding eigenvectors. The eigenvalues represent the variance along each principal component direction, reflecting their relative importance, while the eigenvectors indicate the directions of the principal components.

4. Selection of Principal Components: Typically, the first *k* principal components are selected based on the magnitude of the eigenvalues to achieve dimensionality reduction. The selection criterion can be based on a cumulative contribution rate reaching a predetermined threshold.

5. Data Transformation: The original data are projected onto the new subspace defined by the selected eigenvectors, resulting in a reduced-dimensionality dataset.

In order to improve the efficiency of feature extraction and avoid redundant information, Principal Component Analysis (PCA) was applied to the extracted statistical features. Before performing PCA, all feature parameters were normalized to eliminate the influence of different dimensions. The principal components were then selected according to the cumulative explained variance. A total of 34 statistical parameters were extracted from the PRPD patterns, including phase-related, magnitude-related, and distribution-related features. In this study, all 34 extracted features were directly used as the input of the PCA model without prior feature filtering. PCA was then applied to reduce the dimensionality of the original feature set and to eliminate redundancy among the parameters while preserving the main information contained in the dataset.

In this study, the first five principal components were retained, which together explained more than 90% of the total variance of the original dataset. Therefore, the dimensionality of the feature set was reduced while most of the original information was preserved. The reduced feature set was subsequently used as the input for the partial discharge pattern recognition model. The characteristics of PCA make it a powerful tool for high-dimensional data exploration, noise reduction, and compression, thereby providing a solid theoretical foundation for the construction of fault diagnosis models in subsequent sections.

### 3.2. Multi-Model Voting Fusion Algorithm

The pattern recognition of aging stages in oil-impregnated insulating paper enables the precise determination of the equipment’s current aging condition. The recognition process is characterized by complex aging feature parameters, a nonlinear mapping between features and aging stages, numerous environmental interferences, and the presence of noise superposition and coupling in the feature signals, making the task challenging. When relying on a single model for pattern recognition, it may fail to capture all the intricate details, and its accuracy is difficult to further improve. Under such circumstances, more sophisticated, ensemble-based models can be introduced. By integrating multiple distinct models on top of a single-model framework, the limitations of individual models in specific aspects can be mitigated, thereby enhancing the overall performance of the system.

Linear model fusion is a commonly used ensemble approach, where the results of multiple linear models are combined linearly to produce a more accurate final outcome. Voting-based methods are widely employed for classification tasks, where the outputs of multiple base classifiers are aggregated through a voting mechanism to determine the final class label. Voting methods leverage the diversity of multiple models: when different models provide conflicting predictions for the same class, the final result is more likely to approximate the true outcome, thus improving accuracy.

Voting can be categorized into hard voting and soft voting. In hard voting, each base classifier assigns a discrete class label to each sample, and the final classification is determined according to the majority rule, i.e., the class receiving the most votes is selected as the final result [26]. Hard voting is suitable when the base classifiers output discrete labels. In soft voting, each base classifier provides a probability or confidence score for each class, and the final classification is obtained based on the weighted average of these probabilities [27]. Soft voting enhances hard voting by assigning weights to different classifiers according to their predictive performance, with models exhibiting higher accuracy receiving greater weight in the final decision.

**Figure 10:**
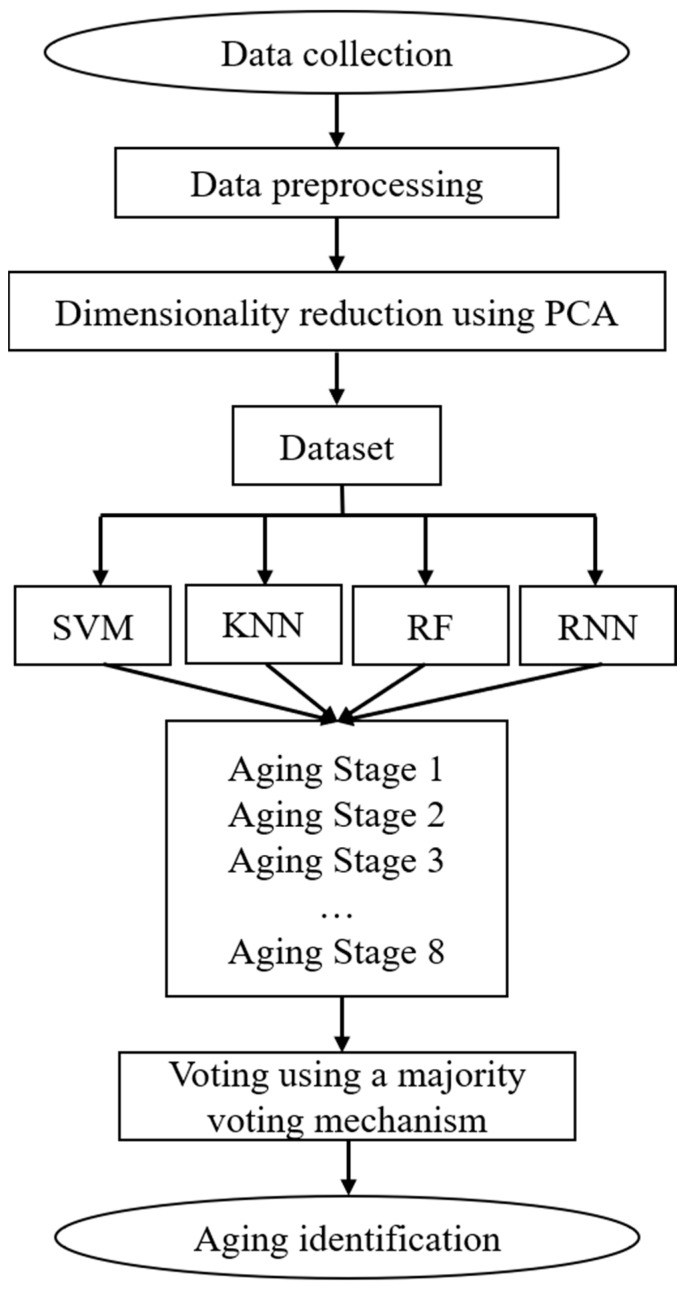
Basic Framework of the Voting-Based Method.

The basic framework of the voting method is illustrated in Figure 10. Initially, appropriate classifiers are selected, and each classifier is trained using the training dataset. Subsequently, the trained classifiers predict the outcomes for the testing dataset, generating individual classification results. Finally, the predictions from all classifiers are aggregated through either majority voting or weighted voting, and the voting decision is taken as the final output of the ensemble classifier.

In the voting fusion model, the weight assigned to each classifier represents its contribution to the final classification result. Classifiers with a greater influence on the final outcome are assigned higher weights, while those with lesser influence receive lower weights. The discriminant function for the output value *y* can be expressed as:

(4) $$y=\sum_{i=1}^{n}w_{i}V_{k}$$

(5) $$w_{i}=\frac{1}{T}$$

In the equation, *i* = 1, 2, …, *n*. denotes the *i*-th classifier’s vote, *w*_*i*_ represents the weight of the vote, and *T* is the total number of base learners participating in the voting process.

The voting fusion (VF) approach for pattern recognition is based on the principle of “strong base model fitting and robust ensemble integration.” Initially, four classical individual learning algorithms—Support Vector Machine (SVM), Random Forest (RF), k-Nearest Neighbors (KNN), and Artificial Neural Network (ANN)—are independently trained and applied to model the partial discharge data. For each individual model, hyperparameter optimization was performed to improve classification performance. Specifically, for the SVM model, the kernel function and penalty parameter (C) were optimized; for the RF model, the number of trees and maximum tree depth were tuned; for the KNN model, the number of nearest neighbors (k) and distance metric were optimized; and for the ANN model, the number of hidden layers, number of neurons, and learning rate were adjusted. The optimal hyperparameters were determined using a cross-validation strategy.

Subsequently, a majority voting strategy was used to integrate the predictions from these individual models, producing the final decision. This approach enables high-precision and high-robustness identification of the aging stages of ester-based oil-impregnated insulating paper. The method effectively mitigates the limitations of single algorithms and enhances recognition stability under complex operating conditions.

### 3.3. Identification Results of Oil-Impregnated Insulating Paper Aging Stages

After data processing and model construction, the classification performance of the SVM, RF, KNN, and ANN models for oil-impregnated insulation paper at different aging stages was evaluated using a 4-fold cross-validation strategy. The dataset was randomly divided into four subsets; three subsets were used for training and the remaining subset was used for validation, and the process was repeated four times to ensure the stability and reliability of the recognition results. The Voting Fusion strategy was then applied by combining the outputs of the four individual models using a majority voting mechanism to obtain the final aging stage identification result.

**Figure 11:**
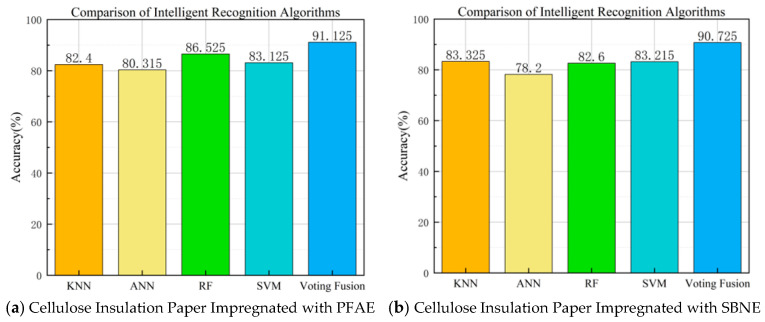
Average Fault Recognition Accuracy of Different Models Based on 4-fold Cross-validation.

During the thermal aging experiment, the oil-impregnated insulation paper underwent eight aging stages, and 40 partial discharge samples were extracted at each stage. The denoised partial discharge signals were directly used as the input to the ANN model. In contrast, 34 statistical features were extracted from the PRPD patterns, and the feature set after PCA dimensionality reduction was used as the input for the SVM, RF, and KNN models. The recognition rates of the individual models and the Voting Fusion model are presented in Figure 11, where the results represent the average recognition accuracy obtained from the 4-fold cross-validation.

As shown in Figure 11a,b, the recognition accuracy of the Voting Fusion algorithm exceeded 90% for both types of oil-impregnated insulation paper. Among the individual algorithms, the ANN consistently exhibited the lowest recognition accuracy, while the other single models demonstrated relatively stable performance. However, their precision was still inferior compared to the Voting Fusion approach. The high accuracy achieved by the Voting Fusion strategy highlights the advantages of multi-model integration, as it combines the strengths of individual algorithms—such as the deployment convenience of KNN and the nonlinear fitting capability of ANN—while mitigating the limitations inherent to single models, thereby enhancing robustness. From an engineering perspective, the Voting Fusion approach provides a reliable and accurate method for identifying the aging stages of ester-based oil-impregnated insulating paper.

**Figure 12:**
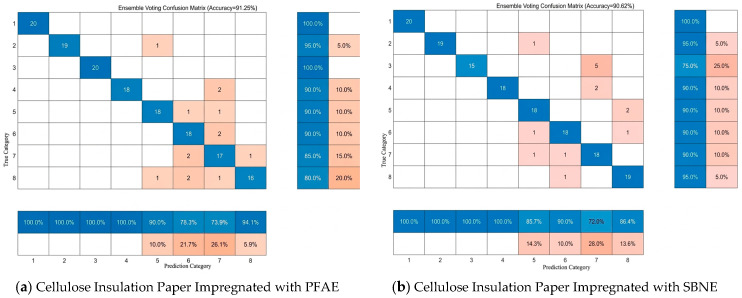
Recognition Accuracy Across Different Aging Stages.

The 320 sample datasets for each type of ester-based insulating oil were divided into eight categories, corresponding to the eight aging stages of each oil type (Categories 1–8 represent aging times of 0 h, 12 h, 24 h, 72 h, 168 h, 312 h, 504 h, and 768 h, respectively). Figure 12 illustrate the prediction results of the Voting Fusion algorithm for the different aging stages of the ester-based insulating oils. A prediction is considered correct when the predicted class matches the true class. From these figures, the recognition rates of the Voting Fusion algorithm for each aging stage of the ester-based insulating oils can be directly observed.

When the sample size is sufficiently large, the advantages of the Voting Fusion algorithm become more pronounced, resulting in significantly improved accuracy. The recognition rates for different aging stages of the ester-based insulating oils ranged from 75% to 100%, with the average recognition rates for both types of oil-impregnated insulating paper exceeding 90%. By integrating the strengths of multiple individual models, the Voting Fusion algorithm effectively reduces misclassification and enhances the overall correct recognition rate.

## 4. Conclusions

This study investigates the degradation mechanism of cellulose in oil-impregnated insulating paper under electro–thermal stress from a polymer science perspective and establishes a laboratory-based framework for characterizing insulation aging using partial discharge (PD) features. By correlating molecular chain scission, microstructural evolution, and macroscopic electrical response, a method for evaluating the structural integrity of cellulose polymers under combined electro–thermal stress is proposed.

The degradation of cellulose exhibits stage-dependent depolymerization behavior. The rapid decrease in the degree of polymerization at the early aging stage indicates severe molecular chain scission in the amorphous regions, while the degradation rate gradually decreases at later stages due to the higher stability of the crystalline regions. Scanning electron microscopy observations further confirm progressive morphological degradation of the fiber network, including fiber separation, distortion, and fracture, which is consistent with the DP degradation behavior. In addition, the evolution of PD characteristics is closely related to cellulose molecular degradation, indicating that discharge behavior is strongly influenced by polymer chain length and structural integrity.

Statistical features extracted from phase-resolved PD patterns were analyzed using principal component analysis to construct a feature space for degradation characterization. A multi-model fusion strategy was further used to identify different degradation stages. The results demonstrate that PD characteristics can serve as effective macroscopic indicators of cellulose degradation and provide a polymer-oriented understanding of aging in natural polymer insulation systems.

Although this study provides a polymer-based interpretation of cellulose degradation under electro–thermal stress, some limitations should be noted. The experiments were conducted under laboratory accelerated aging conditions without validation using real transformer data. Future work will focus on verifying the proposed PD-based characterization method in practical insulation systems and under more complex operating conditions.

## Figures and Tables

**Table 1:** Basic performance parameters of insulating oil.

Parameter	SBNE Insulating Oil	PFAE Insulating Oil
Density (20 °C, g/cm^3^)	0.90	0.86
Moisture Content (ppm)	154.6	237.4
Kinematic Viscosity (40 °C, mm^2^/s)	30.4	5.06
tan δ (90 °C)	0.00418	0.0239
Acid Value (mg KOH/g)	0.028	0.005
Flash Point (°C)	326	186
Breakdown Voltage (2.5 mm, kV)	76.7	81

**Table 2:** Calculated k_1_ and k_2_ values of the two insulating oils based on the kinetic model.

**Type Of Oil-Impregnated Cellulose Paper**	**k_1_**	**k_2_**	**R^2^**	**RMSE**
**Cellulose Insulation Paper Impregnated with SBNE Insulating Oil**	3.11 × 10^−3^	4.9138 × 10^−6^	0.9085	2.4042 × 10^−4^
**Cellulose Insulation Paper Impregnated with PFAE Insulating Oil**	2.81 × 10^−3^	8.0647 × 10^−6^	0.7777	2.0715 × 10^−4^

**Table 3:** Variation in the inception voltage of oil-impregnated insulating paper with aging time.

Aging Time (h)		0	12	24	72	168	312	504	768
**Inception Voltage (kV)—Cellulose Insulation Paper Impregnated with SBNE Oil**	**Average Value**	15.11	14.32	10.69	12.43	13.15	16.52	18.11	18.45
	**Standard Deviation**	0.487	0.306	0.586	0.490	0.410	0.268	0.611	0.413
**Inception Voltage (kV)—** **Cellulose Insulation Paper Impregnated with PFAE Oil**	**Average Value**	14.91	14.37	14.88	13.51	14.66	15.22	14.94	13.10
	**Standard Deviation**	0.487	0.502	0.438	0.391	0.480	0.377	0.320	0.369

**Table 4:** Variation in the breakdown voltage of oil-impregnated insulating paper with aging time.

Aging Time (h)		0	12	24	72	168	312	504	768
**Breakdown Voltage (kV)—Cellulose Insulation Paper Impregnated with SBNE Oil**	**Average Value**	8.54	10.45	7.97	8.32	6.26	8.78	8.81	8.63
	**Standard Deviation**	0.693	0.350	0.580	0.964	0.317	0.634	0.565	0.666
**Breakdown Voltage (kV)—Cellulose Insulation Paper Impregnated with PFAE Oil**	**Average Value**	8.52	9.23	9.15	7.15	9.14	9.29	9.15	6.88
	**Standard Deviation**	0.556	0.377	0.506	0.516	0.377	0.524	0.369	0.296

**Table 5:** Statistical Features of Two-Dimensional Patterns.

Two-Dimensional Patterns	$S_{k}$	Ku	Pe	I	L	FC	VF	cc	$m_{cc}$	Q
$\varphi -{Q_{\max}}^{+}$	$S_{k1}$	$Ku_{1}$	$Pe_{1}$	$I_{1}$	L	FC	$VF_{1}$	$cc_{1}$	$m_{cc1}$	$Q_{1}$
$\varphi -{Q_{\max}}^{-}$	$S_{k2}$	$Ku_{2}$	$Pe_{2}$							
$\varphi -{Q_{ave}}^{+}$	$S_{k3}$	$Ku_{3}$	$Pe_{3}$	$I_{2}$	–	–	$VF_{2}$	$cc_{2}$	$m_{cc2}$	$Q_{2}$
$\varphi -{Q_{ave}}^{-}$	$S_{k4}$	$Ku_{4}$	$Pe_{4}$							
$\varphi -n^{+}$	$S_{k5}$	$Ku_{5}$	$Pe_{5}$	$I_{3}$	–	–	–	$cc_{3}$	$m_{cc3}$	$Q_{3}$
$\varphi -n^{-}$	$S_{k6}$	$Ku_{6}$	$Pe_{6}$							

**Table 6:** Feature Parameters of Cellulose Insulation Paper Impregnated with SBNE Oil.

Aging Time/h	0	12	24	72	168	312	504	768
**Positive Half-Cycle *S_k_*** **_1_**	0.377	0.207	0.183	0.112	0.128	0.492	0.505	0.448
**Negative Half-Cycle *S_k_*** **_2_**	0.610	0.066	0.053	0.091	0.098	0.294	0.291	0.324
**Modified Cross-Correlation Coefficient *m_cc_***	0.675	0.299	0.027	0.127	0.237	0.495	0.645	0.619
**Margin Factor *L***	12.178	11.338	13.168	5.514	8.081	15.243	12.215	17.955
**Pulse Factor *I***	476.266	482.652	405.854	381.738	416.268	513.458	499.975	523.045
**Center Frequency *FC***	8.584	7.975	7.903	4.884	6.933	10.072	7.653	10.750

**Table 7:** Feature Parameters of Cellulose Insulation Paper Impregnated with PFAE Oil.

Aging Time/h	0	12	24	72	168	312	504	768
**Positive Half-Cycle *S_k_*** **_1_**	0.369	0.192	0.301	0.131	0.155	0.221	0.189	0.129
**Negative Half-Cycle *S_k_*** **_2_**	0.384	0.231	0.441	0.093	0.234	0.261	0.212	0.061
**Modified Cross-Correlation Coefficient *m_cc_***	0.693	0.552	0.659	0.125	0.474	0.605	0.569	0.040
**Margin Factor *L***	13.291	11.133	11.450	9.029	8.449	10.944	9.496	6.701
**Pulse Factor *I***	465.500	444.077	440.474	414.373	424.508	443.507	434.654	349.777
**Center Frequency *FC***	9.156	8.533	8.551	7.747	6.995	8.423	7.591	5.936

## Data Availability

Data are contained within the article.
